# Nanometrology: Absolute Seebeck coefficient of individual silver nanowires

**DOI:** 10.1038/s41598-019-56602-9

**Published:** 2019-12-30

**Authors:** M. Kockert, D. Kojda, R. Mitdank, A. Mogilatenko, Z. Wang, J. Ruhhammer, M. Kroener, P. Woias, S. F. Fischer

**Affiliations:** 1 Novel Materials Group, Humboldt-Universität zu Berlin, Newtonstraße 15, 12489 Berlin, Germany; 2 Ferdinand-Braun-Institut, Leibniz-Institut für Höchstfrequenztechnik, Gustav-Kirchhoff-Straße 4, 12489 Berlin, Germany; 3 Laboratory for Design of Microsystems, University of Freiburg - IMTEK, Georges-Köhler-Allee 102, 79110 Freiburg, Germany

**Keywords:** Characterization and analytical techniques, Electronic properties and materials, Electronic devices, Nanowires

## Abstract

Thermoelectric phenomena can be strongly modified in nanomaterials. The determination of the absolute Seebeck coefficient is a major challenge for metrology with respect to micro- and nanostructures due to the fact that the transport properties of the bulk material are no more valid. Here, we demonstrate a method to determine the absolute Seebeck coefficient *S* of individual metallic nanowires. For highly pure and single crystalline silver nanowires, we show the influence of nanopatterning on *S* in the temperature range between 16 K and 300 K. At room temperature, a nanowire diameter below 200 nm suppresses *S* by 50% compared to the bulk material to less than *S* = 1 *μ*VK^−1^, which is attributed to the reduced electron mean free path. The temperature dependence of the absolute Seebeck coefficient depends on size effects. Thermodiffusion and phonon drag are reduced with respect to the bulk material and the ratio of electron-phonon to phonon-phonon interaction is significantly increased.

## Introduction

Bulk silver has the highest electrical and thermal conductivity of all metals^1^ and well-known thermoelectric properties^2–6^. For this reason, it is a widely used material in the electronics industry. In recent years, there has been a great interest in micro- and nanostructures of silver, which are suitable for touch screens, solar cells and batteries^7–9^. A small absolute Seebeck coefficient is important for interconnects in low-noise electronics. While the thermoelectric properties of metallic thin films have been studied^5,10–16^, it is very challenging to determine these of individual metallic nanowires^17–21^.

In general, the thermovoltage is sensitive to structural defects, metal grain structure and surface passivation, e.g. for polycrystalline silver nanowires, the transport properties are therefore reduced compared to the bulk material^18^. Individual single crystalline silver nanowires are well suited to study size effects^19^.

However, the absolute Seebeck coefficient of individual silver nanowires remains an open issue. In particular, the determination of the absolute Seebeck coefficient of an individual metallic nanowire requires the knowledge of the absolute Seebeck coefficient of the micro- and nanopatterned thermoelectric reference material. For such, it has been shown that the Seebeck coefficient is size-dependent^10–12^.

Here, high-purity and single crystalline nanowires serve as a model system to investigate the influence of nanopatterning on the absolute Seebeck coefficient *S*. The examined silver nanowires show no change of the crystal structure, the electron density and the Debye temperature compared to bulk silver^19^. The essential difference between the silver nanowires and bulk silver is that the electron mean free path is comparable to the diameter of the nanowires^19^.

Thermodiffusion and phonon drag are the two main contributions to the absolute Seebeck coefficient^11,12,22^. The thermodiffusion of charge carriers in the material caused by a temperature difference can be described by Mott’s formula^11,12,22,23^. The phonon drag arises from the interaction between electrons and phonons^11,12,22,24^.

Here, we demonstrate a high precision method to measure ultra-low absolute Seebeck coefficients of individual metallic nanowires. We present a model for the temperature dependence of *S* and discuss the influence of electron-phonon and phonon-phonon interaction on the absolute Seebeck coefficient of silver nanowires and bulk silver. The thermoelectric properties correlate with size effects and lead to the vanishing behavior of the absolute Seebeck coefficient of the silver nanowires.

## Results

### Structural properties

**Figure 1:**
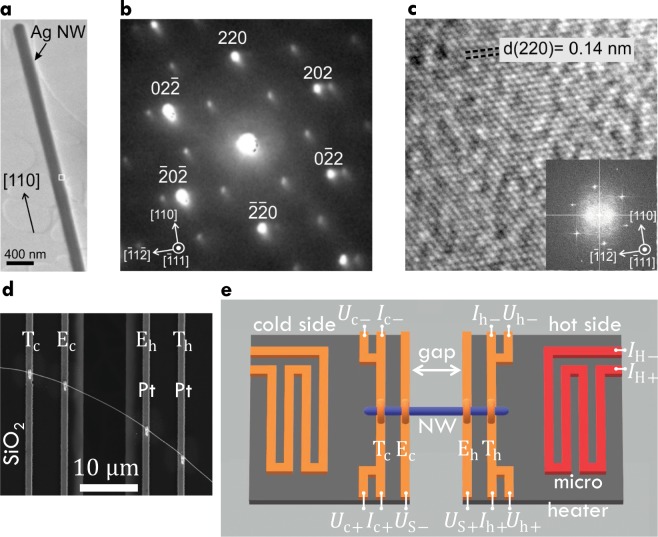
Transmission electron microscopy, selected area electron diffraction, scanning electron microscopy images of silver nanowires and a sketch of the measurement area of the thermoelectric nanowire characterization platform. (**a**) Transmission electron microscopy image of a silver nanowire placed on a carbon film showing the growth direction of the nanowire. (**b**) Selected area electron diffraction confirms the face centered cubic structure of the silver nanowire viewed along one of the 〈111〉 directions. Additional diffraction spots, which are not indexed in the image correspond to a 〈110〉 direction and appear due to the presence of a twin in the nanowire^19^. (**c**) High-resolution transmission electron microscopy image showing atomic planes. The inset shows the fast Fourier transformation. (**d**) Scanning electron microscopy image of a silver nanowire (NW 3) contacted on platinum (Pt) conduction lines of the thermoelectric nanowire characterization platform by electron beam-induced deposition. ${{\rm{T}}}_{{\rm{c}}}$ and ${{\rm{T}}}_{{\rm{h}}}$ are the thermometer electrodes of the cold and hot side, respectively. ${{\rm{E}}}_{{\rm{c}}}$ and ${{\rm{E}}}_{{\rm{h}}}$ are the thermovoltage electrodes of the cold and hot side, respectively. (**e**) Sketch of the measurement area of the platform. An electron transparent gap divides the measurement area into two sides. In order to measure the thermovoltage of the silver nanowire (NW) (blue) relative to platinum conduction lines ${{\rm{E}}}_{{\rm{c}}}$ and ${{\rm{E}}}_{{\rm{h}}}$ (orange). A heating current ${I}_{{\rm{H}}}$ is applied at the micro heater (red), which creates a temperature difference along the nanowire that can be calculated by four-terminal resistance measurements of the thermometers ${{\rm{T}}}_{{\rm{c}}}$ and ${{\rm{T}}}_{{\rm{h}}}$ (orange) for the cold and hot side, respectively.

The crystallinity of the individual silver nanowires was confirmed by transmission electron microscopy. Figure 1a shows a silver nanowire placed on a lacey carbon film. The growth direction is along [110]. Selected area electron diffraction analysis and high-resolution transmission electron microscopy confirm the face centered cubic structure as given in Fig. 1b,c, respectively. The structural and chemical properties were discussed in detail previously^19^.

### Electrical properties

**Figure 2:**
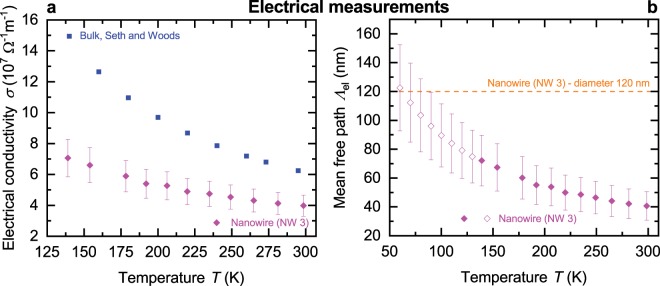
Electrical conductivity and electron mean free path as a function of bath temperature. (**a**) Temperature-dependent electrical conductivity *σ* of a silver nanowire (NW 3) and bulk silver^29^. (**b**) Electron mean free path ${\Lambda }_{{\rm{el}}}$ as a function of the bath temperature *T*. The orange dashed line represents the diameter of a silver nanowire (NW 3). At low bath temperatures, the electron mean free path is mainly limited by the diameter of the nanowire. For $T > 130\,{\rm{K}}$, the electron mean free path (filled symbols) was calculated from the measured electrical conductivity as given in (**a**). For $T\le 130\,{\rm{K}}$, the electron mean free path (open symbols) was calculated from the extrapolated electrical resistance (electrical conductivity) that has been determined by the Bloch-Grüneisen formula.

A thermoelectric nanowire characterization platform (TNCP)^19,25,26^ was used in order to determine the electrical conductivity and the Seebeck coefficient of the silver nanowires. Electron beam-induced deposition contacts were prepared to improve the electrical and mechanical connection between the nanowire and the measurement platform. A scanning electron microscopy image of a silver nanowire (NW 3) on top of the platinum conduction lines of the TNCP is depicted in Fig. 1d. A schematic of the measurement setup is given in Fig. 1e. For extensive descriptions of the TNCP and length scales, see Kojda *et al*.^19,20,25^ Geometry parameters determined by scanning and transmission electron microscopy and as well as the electrical conductivity *σ* determined from four-terminal resistance measurements at room temperature are given in Table 1. The electrical conductivity of the silver nanowires is reduced compared to the bulk material, see Fig. 2a. This can be attributed to size effects, which reduce the electron mean free path in the silver nanowires as shown in Fig. 2b. The results of the electrical measurements are in agreement with Kojda *et al*.^19^.

**Table 1:** Geometry parameters and electrical conductivity.

Sample	*l* (*μ*m)	*d* (nm)	*σ* (10^7^ Ω^−1^ m^−1^)
NW 1	18.9 ± 0.4	190 ± 10	3.3 ± 0.4
NW 2	18.1 ± 0.8	180 ± 5	4.9 ± 0.4
NW 3	16.0 ± 0.7	120 ± 10	4.0 ± 0.7
NW 4	15.0 ± 1.0	120 ± 20	—

Overview of length *l* and diameter *d* of each silver nanowire determined by scanning and transmission electron microscopy. In addition, the electrical conductivity *σ* at room temperature is given.

### Relative Seebeck coefficient

**Figure 3:**
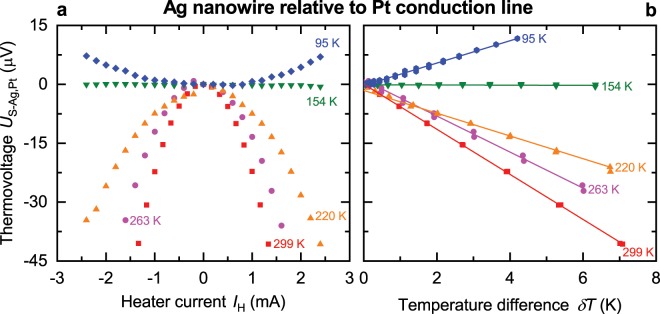
Thermovoltage as a function of heater current and temperature difference of a silver (Ag) nanowire. (**a**) Thermovoltage ${U}_{{\rm{S}}-{\rm{Ag}},{\rm{Pt}}}$ of a silver nanowire (NW 3) as a function of the heater current ${I}_{{\rm{H}}}$. (**b**) Thermovoltage ${U}_{{\rm{S}}-{\rm{Ag}},{\rm{Pt}}}$ of the silver nanowire versus temperature difference $\delta T$. The slope of the fitted solid lines yields the relative Seebeck coefficient.

The temperature-dependent Seebeck coefficient ${S}_{{\rm{Ag}},{\rm{Pt}}}$ of individual silver nanowires relative to 200 nm thick platinum conduction lines was measured between bath temperatures of 16 K and room temperature. The thermovoltage ${U}_{{\rm{S}} \mbox{-} {\rm{Ag}},{\rm{Pt}}}$ as a function of the micro heater current ${I}_{{\rm{H}}}$ is presented for a selection of bath temperatures in Fig. 3a. Its parabolic behavior confirms the presence of the thermoelectric effect. Figure 3b shows the thermovoltage as a function of the temperature difference. The temperature difference $\delta T$ between the hot and the cold side of the silver nanowires was calculated by the change of four-terminal-resistance thermometers due to the variation of the power of the micro heater. The slope of the function ${U}_{{\rm{S}} \mbox{-} {\rm{Ag}},{\rm{Pt}}}(\delta T)$ gives the Seebeck coefficient ${S}_{{\rm{Ag}},{\rm{Pt}}}$ of the silver nanowires with respect to the platinum conduction lines

1 $${S}_{{\rm{Ag}},{\rm{Pt}}}={S}_{{\rm{Ag}}}-{S}_{{\rm{Pt}}}=-\,\frac{{\rm{d}}{U}_{{\rm{S}} \mbox{-} {\rm{Ag}},{\rm{Pt}}}}{{\rm{d}}\delta T}\mathrm{}.$$

**Figure 4:**
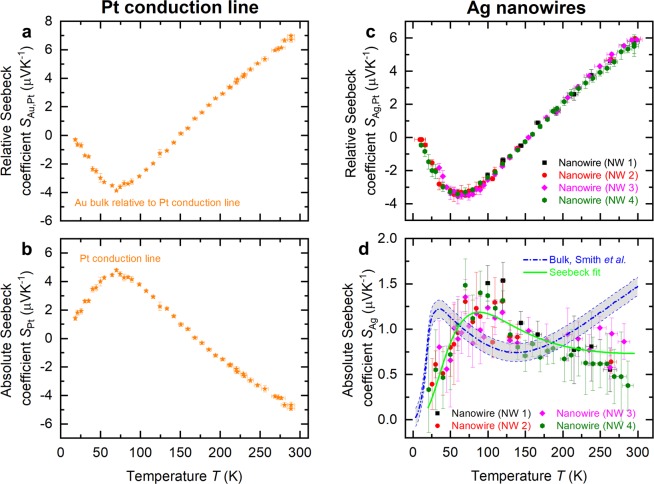
Relative and absolute Seebeck coefficent of a platinum (Pt) conduction line and silver (Ag) nanowires. (**a**) Seebeck coefficient ${S}_{{\rm{Au}},{\rm{Pt}}}$ of a bulk gold wire relative to a thin platinum conduction line as a function of the bath temperature *T*. (**b**) Absolute Seebeck coefficient ${S}_{{\rm{Pt}}}$ of the platinum conduction line versus bath temperature *T*. (**c**) Relative Seebeck coefficient ${S}_{{\rm{Ag}},{\rm{Pt}}}$ of four individual silver nanowires (NW 1, NW 2, NW 3 and NW 4) relative to the platinum conduction line as a function of bath temperature *T*. (**d**) Absolute Seebeck coefficient ${S}_{{\rm{Ag}}}$ of the silver nanowires versus bath temperature $T$. The thick dashed blue line indicates the absolute Seebeck coefficient of bulk silver. The gray shaded area marks the uncertainty of the bulk Seebeck coefficient. Temperature-dependent fit (see Eq. 9) of ${S}_{{\rm{Ag}}}$ of the silver nanowires is depicted as a solid green line.

The temperature dependence of the relative Seebeck coefficient, Fig. 4c, agrees well for different individual silver nanowires. ${S}_{{\rm{Ag}},{\rm{Pt}}}$ is decreasing with decreasing bath temperature, a sign reversal occurs at 150 K. Below 50 K, the relative Seebeck coefficient tends to zero with decreasing bath temperature.

### Absolute Seebeck coefficient

In order to obtain the absolute Seebeck coefficient of a silver nanowire, which is given by

2 $${S}_{{\rm{Ag}}}={S}_{{\rm{Ag}},{\rm{Pt}}}+{S}_{{\rm{Pt}}},$$

the absolute Seebeck coefficient of the platinum conduction line ${S}_{{\rm{Pt}}}$ is required. ${S}_{{\rm{Pt}}}$ was determined in a separate experiment^10^ by measuring a bulk gold wire with known absolute Seebeck coefficient relative to the thin platinum conduction line. See also Supplementary Information S3. A detailed discussion of the absolute Seebeck coefficient of thin platinum films and the effects of heat treatment on the transport properties is given in reference^10^. The relative and absolute Seebeck coefficients of the reference platinum film are given in Fig. 4a,b.

The relative and absolute Seebeck coefficients of the silver nanowires versus bath temperature are given in Fig. 4c,d, respectively. The reference value of the absolute Seebeck coefficient of bulk silver^3,6^ is plotted as a blue line in Fig. 4d. The gray shaded area around the blue line defines the uncertainty of the Seebeck coefficient due to several data given in the literature^2–4,6^.

Our results show that the absolute Seebeck coefficient of the silver nanowires is reduced compared to the bulk material and exhibits only a weak temperature dependence in the temperature range from 200 K up to 300 K. In this regime, the absolute Seebeck coefficient of bulk silver exhibits a linear temperature dependence. In the temperature range from 200 K down to 100 K, the absolute Seebeck coefficient of the silver nanowires is increasing with decreasing bath temperature and reaches the maximum at 88 K. From 150 K down to 100 K the Seebeck coefficient of the silver nanowires is larger than the corresponding bulk Seebeck coefficient. The maximum Seebeck coefficient of bulk silver can be observed at 35 K. The phonon drag peak of the silver nanowires is shifted towards higher bath temperatures compared to the bulk material. The absolute Seebeck coefficient of the silver nanowires and of bulk silver tend to zero with decreasing bath temperature after reaching their maximum value.

## Discussion

The electron mean free path ${\Lambda }_{{\rm{el}}}$ was determined from the change of the electrical conductivity of the nanowire compared to the bulk material. Under the assumption that scattering events reduce the mean free path, it can be described and related to Matthiessen rule^10,19^.

3 $${\Lambda }_{{\rm{el}},{\rm{nw}}}{(T)}^{-1}={\Lambda }_{{\rm{el}},{\rm{b}}}{(T)}^{-1}+{\Lambda }_{{\rm{el}},{\rm{s}}}^{-1}$$

is the inverse nanowire electron mean free path, ${\Lambda }_{{\rm{el}},{\rm{b}}}{(T)}^{-1}$ is the inverse mean free path of the bulk material and ${\Lambda }_{{\rm{el}},{\rm{s}}}^{-1}$ is the inverse temperature-independent scattering length of the electrons due to surface scattering.

4 $${\Lambda }_{{\rm{el}},{\rm{nw}}}(T)=\frac{{\sigma }_{{\rm{nw}}}(T)}{{\sigma }_{{\rm{nw}}}({\rm{RT}})}{\Lambda }_{{\rm{el}},{\rm{nw}}}({\rm{RT}})$$

and

5 $${\Lambda }_{{\rm{el}},{\rm{b}}}(T)=\frac{{\sigma }_{{\rm{b}}}(T)}{{\sigma }_{{\rm{b}}}({\rm{RT}})}{\Lambda }_{{\rm{el}},{\rm{b}}}({\rm{RT}})$$

are the electron mean free paths of the nanowire and of the bulk material, respectively. For bulk silver, a value of ${\Lambda }_{{\rm{el}},{\rm{b}}}({\rm{RT}})=53\,{\rm{nm}}$ is reported at room temperature^27^.

For a nanowire, the electron mean free path was determined from the measured electrical resistance (electrical conductivity) in the temperature range from room temperature down to $T=140\,{\rm{K}}$. For $T\le 130\,{\rm{K}}$, the electron mean free path was calculated from the extrapolated electrical resistance (electrical conductivity) that has been determined by the Bloch-Grüneisen formula. Figure 2b shows the electron mean free path (NW 3) as a function of the bath temperature, it is in the order of the nanowire diameter. Therefore, the enhanced surface scattering leads to a reduced electrical conductivity of the silver nanowires compared to the bulk material^19^.

The absolute Seebeck coefficient *S* is the sum of the thermodiffusion ${S}_{{\rm{diff}}}$ and the phonon drag ${S}_{{\rm{ph}}}$ contributions^11,12,22^, $S={S}_{{\rm{diff}}}+{S}_{{\rm{ph}}}$. For temperatures higher than the Debye temperature ($T > {\Theta }_{{\rm{D}}}$), the absolute Seebeck coefficient is dominated by phonon-phonon scattering events^22^, which leads to a linear temperature dependence and can be described by Mott’s formula^10–12,23^.

The phonon drag effect is an indicator of the strength of the electron-phonon interaction and has a strong influence on *S* of metals below the Debye temperature^11,12,22^. It is connected with the specific heat of the phonons^12,22^

6 $${C}_{{\rm{ph}}}(T)=9n{k}_{{\rm{B}}}{(\frac{T}{{\Theta }_{{\rm{D}}}})}^{3}\,{\int }_{0}^{\frac{{\theta }_{{\rm{D}}}}{T}}\,\frac{{x}^{4}\,\exp (x)}{{(\exp (x)-1)}^{2}}{\rm{d}}x$$

with the number of charge carriers per volume *n* and $x=\frac{\hslash {\omega }_{{\rm{D}}}}{{k}_{{\rm{B}}}T}=\frac{{\Theta }_{{\rm{D}}}}{T}$ that is given by the reduced Planck constant $\hslash$, the Debye frequency ${\omega }_{{\rm{D}}}$ and the Boltzmann constant ${k}_{{\rm{B}}}$.

The phonon drag contribution to *S* is given by^10,22^

7 $${S}_{{\rm{ph}}}={F}_{{\rm{ph}}}\frac{{C}_{{\rm{ph}}}(T)}{3ne}\gamma ={F}_{{\rm{ph}}}\frac{{C}_{{\rm{ph}}}(T)}{3ne}\frac{{\tau }_{{\rm{pp}}}}{{\tau }_{{\rm{pp}}}+{\tau }_{{\rm{ep}}}}.$$

Therefore, the phonon-phonon scattering time ${\tau }_{{\rm{pp}}}$ and the scattering time of the electron-phonon interaction ${\tau }_{{\rm{ep}}}$ has to be taken into account^22^. The temperature dependence of the electron-phonon and phonon-phonon interaction can be described by the *γ*-factor

8 $$\gamma =\frac{1}{1+\frac{{\tau }_{{\rm{ep}}}}{{\tau }_{{\rm{pp}}}}}=\frac{1}{1+{F}_{\tau }T\,\exp (\,-\,\frac{{\Theta }_{{\rm{D}}}}{T})}\mathrm{}.$$

The relation $\frac{{\tau }_{{\rm{ep}}}}{{\tau }_{{\rm{pp}}}}={F}_{\tau }T\,\exp (\,-\,\frac{{\Theta }_{{\rm{D}}}}{T})$ was adapted from^12,28^, assuming that the phonon-phonon interaction becomes dominant at high temperatures^12,28^. Hence, the temperature dependence of the absolute Seebeck coefficient is given by

9 $$S(T)={F}_{{\rm{diff}}}\frac{T}{{\Theta }_{{\rm{D}}}}+\frac{{F}_{{\rm{ph}}}{(\frac{T}{{\Theta }_{{\rm{D}}}})}^{3}\,{\int }_{0}^{\frac{{\theta }_{{\rm{D}}}}{T}}\,\frac{{x}^{4}\,\exp (x)}{{(\exp (x)-1)}^{2}}{\rm{d}}x}{1+{F}_{\tau }T\,\exp (\,-\frac{{\Theta }_{{\rm{D}}}}{T})}\mathrm{}.$$

The parameters ${F}_{{\rm{diff}}}$, ${F}_{{\rm{ph}}}$ and ${F}_{\tau }$ include the size effects on behalf of nanopatterning and are discussed below.

The thermodiffusion of charge carriers due to a temperature difference along the silver nanowires is described by the parameter ${F}_{{\rm{diff}}}$. Applying $S(T)$ from Eq. 9 on our measurement data, yields ${F}_{{\rm{diff}},{\rm{NWs}}}=\mathrm{(0.3}\pm \mathrm{0.1)}\,\mu {{\rm{VK}}}^{-1}$ and ${F}_{{\rm{diff}},{\rm{bulk}}}=\mathrm{(1.0}\pm \mathrm{0.1)}\,\mu {{\rm{VK}}}^{-1}$. The parameter ${F}_{{\rm{diff}}}$ of the silver nanowires is reduced compared to the bulk value. This can be attributed to the electron mean free path that is in the order of the nanowire diameter and leads to more surface scattering events compared to the bulk material. Bulk silver exhibits a linear temperature dependence of the Seeebeck coefficient above the Debye temperature (${\Theta }_{{\rm{D}},{\rm{Ag}},{\rm{bulk}}}=215\,{\rm{K}}$^19^), which is not observed for the silver nanowires in the investigated temperature range.

The phonon drag contribution is described by the parameter ${F}_{{\rm{ph}}}$. The fit parameters of bulk silver ${F}_{{\rm{ph}},{\rm{bulk}}}=\mathrm{(12}\pm \mathrm{2)}\,\mu {{\rm{VK}}}^{-1}$ and of the silver nanowires ${F}_{{\rm{ph}},{\rm{NWs}}}=\mathrm{(5.2}\pm \mathrm{0.4)}\,\mu {{\rm{VK}}}^{-1}$ scale the influence of the phonon drag on the Seebeck coefficient. ${F}_{{\rm{ph}},{\rm{NWs}}}$ is reduced compared to ${F}_{{\rm{ph}},{\rm{bulk}}}$. The temperature profile of the phonon drag contribution to the Seebeck coefficient of the silver nanowires is broadened compared to the bulk material. Furthermore, a shift of the phonon drag peak towards higher bath temperatures compared to the bulk material is observed. For temperatures $T > 300\,{\rm{K}}$, the absolute Seebeck coefficient of the silver nanowires is expected to increase linear with increasing bath temperatures according to thermodiffusion contribution.

The electron-phonon vs. phonon-phonon interaction is as follows. The parameter ${F}_{\tau }$ $({F}_{\tau ,{\rm{bulk}}}=\mathrm{(1.2}\pm \mathrm{0.3)}\,{{\rm{K}}}^{-1}$, ${F}_{\tau ,{\rm{NWs}}}=\mathrm{(0.03}\pm \mathrm{0.01)}\,{{\rm{K}}}^{-1})$ gives the ratio of the scattering time of the electron-phonon interaction and of the phonon-phonon interaction and determines the *γ*-factor. ${F}_{\tau ,{\rm{NWs}}}$ is reduced compared to the bulk material.

**Figure 5:**
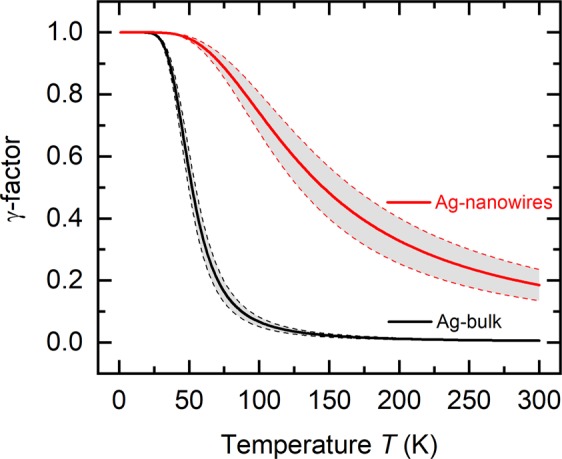
*γ*-factor of silver nanowires and bulk silver. The panel shows the *γ*-factor versus temperature $T$. The *γ*-factor is a function of the electron-phonon and phonon-phonon interaction of the silver nanowires (red line) and bulk silver (black line), respectively. The gray shaded area marks the uncertainty. $\gamma =1$ means that the electron-phonon interaction compared to the phonon-phonon interaction is dominant. $\gamma =0$ means that the phonon-phonon interaction compared to the electron-phonon interaction is dominant.

Figure 5 shows the *γ*-factor of the silver nanowires and bulk silver as a function of the temperature. The *γ*-factor is a number between 0 and 1, which depends on the interaction between phonons and electrons. At low temperatures $T\ll {\Theta }_{{\rm{D}}}$, $\gamma \approx 1$. This means that the temperature dependence of the phonon drag is determined by the specific heat that results in ${S}_{{\rm{ph}}}\propto {T}^{3}$. Phonon-phonon interaction is dominant for $\gamma \approx 0$. This behavior occurs at temperatures $T > {\Theta }_{{\rm{D}}}$, so ${S}_{{\rm{ph}}}\propto \frac{1}{T}$.

In general, the difference between the *γ*-factor of the silver nanowires $({\gamma }_{{\rm{NWs}}})$ and of the bulk material $({\gamma }_{{\rm{bulk}}})$ can be explained by the small diameter of the nanowires and the much larger surface-area-to-volume ratio of the silver nanowires compared to bulk silver.

*γ*_NWs_ is larger than *γ*_bulk_ at room temperature. This can be attributed to a stronger reduction of the thermodiffusion contribution compared to the phonon drag contribution to the absolute Seebeck coefficient of the silver nanowires. For this reason, it can be derived that the electron-phonon interaction has already an influence on the absolute Seebeck coefficient of the silver nanowires at 300 K. Whereas, *γ*_bulk_ is dominated by the phonon-phonon interaction. This is expressed in a significant amount of the thermodiffusion contribution compared to the phonon drag contribution to the absolute Seebeck coefficient of bulk silver.

*γ*_NWs_ and *γ*_bulk_ increase with decreasing temperatures. Below 80 K, the increase of ${\gamma }_{{\rm{NWs}}}$ is less than the increase of ${\gamma }_{{\rm{bulk}}}$ because the influence of the phonon drag on the absolute Seebeck coefficient of the silver nanowires is decreasing. In contrast to the silver nanowires, the influence of the phonon drag on the absolute Seebeck coefficient of bulk silver is increasing in this temperature range.

In this study, we demonstrated a method to determine the absolute Seebeck coefficient of individual metallic nanowires. The detailed analysis showed that both the thermodiffusion and phonon drag contributions to the absolute Seebeck coefficient of individual single crystalline silver nanowires are reduced compared to bulk silver. In particular, the thermodiffusion part is reduced more than the phonon drag part. The reason for this reduction can be attributed to size effects like surface scattering, which lead to an electron mean free path that is comparable to the nanowire diameter. The ratio of the electron-phonon to phonon-phonon interaction is larger in the silver nanowires than in the bulk material. As a consequence of the small diameter of the nanowires, a channeling effect may restrict the phonon-phonon interaction perpendicular to the nanowire growth direction. For this reason, the electron-phonon interaction in the silver nanowires is significant at room temperature. In general, silver, gold and copper have a similar absolute Seebeck coefficient at room temperature as well as a similar temperature dependence^2^. For this reason, the effects of micro- and nanopatterning in these metals should lead to comparable results of the absolute Seebeck coefficient. The possibility of suppressing the absolute Seebeck coefficient by design of nanopatterned metallic interconnects (below $S=1\,\mu {{\rm{VK}}}^{-1}$ at room temperature) may become important for low-noise applications.

## Methods

### Synthesis, placement and contacts

Highly pure and single crystalline silver nanowires were synthesized by the reduction of silver nitrate (purity: $\mathrm{99.9999 \% }$) with ethylene glycol in presence of copper dichloride dihydrate and polyvinylpyrrolidone as described in^19^. The nanowire suspension was immersed to a grooved silicon wafer. Individual nanowires were lifted from the wafer and assembled on a thermoelectric nanowire characterization platform (TNCP) by a mechanical transfer method. As shown, the TNCP features the combined full thermoelectric characterization of an individual nanowire that includes the determination of the electrical conductivity, the thermal conductivity, the Seebeck coefficient, the structural properties, the chemical composition and the morphology^19^. Figure 1e shows a sketch of the TNCP including the micro heater (red), the platinum thermometers ${{\rm{T}}}_{{\rm{c}}}$ and ${{\rm{T}}}_{{\rm{h}}}$ (orange) and the platinum conduction lines for the thermovoltage measurements ${{\rm{E}}}_{{\rm{h}}}$, ${{\rm{E}}}_{{\rm{c}}}$ (orange). The electrical and mechanical connection between the nanowires and the TNCP was performed by electron beam-induced deposition (EBID) as described in^19^. One nanowire with contacts made by EBID is shown in the scanning electron microscopy (SEM) image in Fig. 1d.

### Measurement setup

The Seebeck coefficients of four individual silver nanowires were measured relative to the Seebeck coefficient of platinum conduction lines with a thickness of 200 nm in a flow cryostat in the temperature range between 16 K and room temperature in an ambient helium atmosphere. A temperature difference along the nanowires was applied by one of the micro heaters at certain stabilized bath temperatures *T*. For different heater powers the thermovoltage ${U}_{{\rm{S}} \mbox{-} {\rm{Ag}},{\rm{Pt}}}$ was measured with respect to the cold side of the nanowires. The temperature difference $\delta T$ between the hot side and the cold side of the nanowires was determined by the measured four-terminal resistance change of the calibrated thermometer lines ${{\rm{T}}}_{{\rm{h}}}$ and ${{\rm{T}}}_{{\rm{c}}}$. Then, the relative Seebeck coefficient is defined by

10 $${S}_{{\rm{Ag}},{\rm{Pt}}}=-\,\frac{{\rm{d}}{U}_{{\rm{S}} \mbox{-} {\rm{Ag}},{\rm{Pt}}}}{{\rm{d}}\delta T}\mathrm{}.$$

In the experiments, the heater power is controlled by a Keithley SourceMeter 2401. The thermometer resistances are determined by four terminal measurements performed by a Keithley 6221 and 2182 A. The thermovoltage is measured by a Keithley 2182 A Nanovoltmeter. The measurement configurations are changed by a Keithley 7001 switch matrix system. The absolute Seebeck coefficient of the platinum conduction line was determined within a separate experiment^10^.

## Supplementary information

Supplementary information.
