# Supplementary material for: Nanometrology: Absolute Seebeck coefficient of individual silver nanowires
Source: Sci Rep. 2019 Dec 30;9:20265. doi: 10.1038/s41598-019-56602-9 (PMC6937240; doi:10.1038/s41598-019-56602-9)
Supplement: Supplementary file 1 — Supplementary information. [file 41598_2019_56602_MOESM1_ESM.pdf]

Supplementary Information - Nanometrology: Absolute Seebeck coefficient of individual silver nanowires

M. Kockert,[1] D. Kojda,[1] R. Mitdank,[1] A. Mogilatenko,[2] Z. Wang,[3]
J. Ruhhammer,[3] M. Kroener,[3] P. Woias,[3] and S. F. Fischer[1,*]

[1]*Novel Materials Group, Humboldt-Universität zu Berlin, Newtonstraße 15, 12489 Berlin, Germany*
[2]*Ferdinand-Braun-Institut, Leibniz-Institut für Höchstfrequenztechnik, Gustav-Kirchhoff-Straße 4, 12489 Berlin, Germany*
[3]*Laboratory for Design of Microsystems, University of Freiburg - IMTEK, Georges-Köhler-Allee 102, 79110 Freiburg, Germany*

(Dated: November 22, 2019)

S1: Absolute Seebeck coefficient of bulk silver

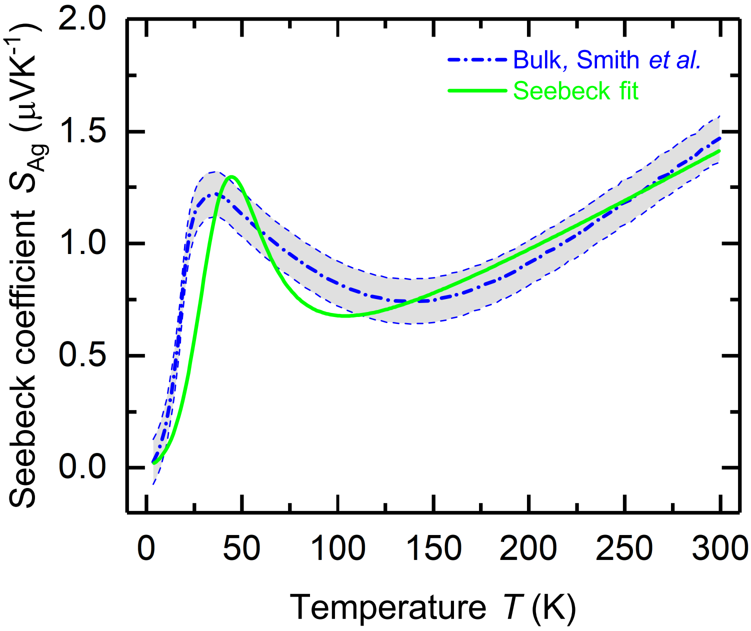

FIG. 1: **Absolute Seebeck coefficent of bulk silver (Ag).** Absolute Seebeck coefficient S_{Ag} of bulk silver versus bath temperature T taken from literature[1]. The thick dashed blue line indicates the absolute Seebeck coefficient of bulk silver. The gray shaded area marks the uncertainty of the bulk Seebeck coefficient. Temperature-dependent fit of S_{Ag} is depicted as a solid green line.

The formula

$$S(T) = F_{\mathrm{diff}}\frac{T}{\Theta_{\mathrm{D}}} + \frac{F_{\mathrm{ph}}\left(\frac{T}{\Theta_{\mathrm{D}}}\right)^3 \int_0^{\frac{\theta_{\mathrm{D}}}{T}} \frac{x^4\exp(x)}{(\exp(x)-1)^2}\mathrm{d}x}{1+F_\tau T\exp\left(-\frac{\Theta_{\mathrm{D}}}{T}\right)}. \quad (1)$$

was applied on the temperature-dependent absolute Seebeck coefficient of bulk silver[1]. The fit parameters are given in table I and are discussed in the main text.

*Correspondence and requests should be addressed to M.K. and S.F.F. (e-mail: kockert@physik.hu-berlin.de, sfischer@physik.hu-berlin.de).

S2: Error analysis

Electrical measurements.

The uncertainty of the electrical conductivity σ of the silver nanowires primarily comes from the determination of the geometry parameters. The diameter d of the silver nanowires was measured by scanning (SEM) and transmission electron microscopy (TEM) at several points along each nanowire. The uncertainty of the diameter results from the resolution limitation of the SEM and TEM investigations and from the nanowire diameter variation and is between 5 nm and 20 nm. The length l of the silver nanowires was measured by scanning electron microscopy. The uncertainty of the length l mainly comes from the size of contact area that is defined by the electron beam-induced deposition contacts. The uncertainty of the length varies between $0.4\,\mu$m and $1.0\,\mu$m. The four-terminal resistance R was determined by linear fits of corresponding I-V curves. The relative uncertainty of R is less than 1 %. Overall, the average relative uncertainty of σ at room temperature is about 12.5 %.

The uncertainty of the electron mean free path Λ of the silver nanowire (NW 3) is mainly set by the uncertainty of the electrical conductivities coming from the silver nanowires and from bulk silver. The electrical conductivity of the silver nanowire was measured from room temperature down to $T = 140\,$K and extrapolated from the electrical resistance by the Bloch-Grüneisen formula for $T \leq 130\,$K. The relative uncertainty of Λ is about 24 %.

Seebeck coefficient.

The relative Seebeck coefficient is determined by the thermovoltage U_{S} and the temperature difference δT that is created by a micro heater. The temperature difference is increased stepwise by applying a heating current I_{H} from zero to $-I_{\mathrm{H,max}}$ and from zero to $+I_{\mathrm{H,max}}$ in equidistant steps. At each step, the thermovoltage is measured ten times and then arithmetically averaged. The uncertainty of the thermovoltage is given by the confidence interval of the measurement results. The relative Seebeck coefficient is given by the mean of the three slopes of the fit lines to the $U_{\mathrm{S}}(0... - I_{\mathrm{H,max}})$ versus $\delta T(0... - I_{\mathrm{H,max}})$, $U_{\mathrm{S}}(0... + I_{\mathrm{H,max}})$ versus $\delta T(0... + I_{\mathrm{H,max}})$ and $U_{\mathrm{S}}(0...-, +I_{\mathrm{H,max}})$ versus $\delta T(0...-, +I_{\mathrm{H,max}})$ plots, respectively. The uncertainty of the relative Seebeck coefficient is determined by the modulus of the largest deviation of the mean value. The average temperature increase by the micro heater determines the uncertainty of the bath temperature T of the temperature-dependent Seebeck coefficient $S(T)$, which is typically less than 5 % of the bath temperature.

The absolute Seebeck coefficient of a calibrated platinum conduction line was used to determine the absolute Seebeck coefficient of the silver nanowires by the following equation,

$$S_{\mathrm{Ag}} = S_{\mathrm{Ag,Pt}} + S_{\mathrm{Pt}}. \tag{2}$$

Only data points of $S_{\mathrm{Ag,Pt}}$ and S_{Pt} at equal bath temperatures (no interpolation) were used to calculate S_{Ag}. The resulting uncertainty of the absolute Seebeck coefficient was determined by propagation of uncertainty.

Seebeck model fit.

Applying formula 1 on our measurement data yields the parameters F_{diff}, F_{ph} and F_{τ}. A best, a maximum and a minimum fit line to the measurement data was used to determine the arithmetic mean of each parameter. The uncertainty was derived from the largest deviation of the mean value. The parameters of the silver nanowires and of the bulk material are given in table I.

Fit parameter	Silver nanowires	Bulk silver
F_{diff} (μVK^{-1})	0.3 ± 0.1	1.0 ± 0.1
F_{ph} (μVK^{-1})	5.2 ± 0.4	12 ± 2
F_{τ} (K^{-1})	0.03 ± 0.01	1.2 ± 0.3

TABLE I: **Fit parameters.** Overview of the fit parameters of the thermodiffusion contribution F_{diff}, the phonon drag contribution F_{ph} and the ratio of the scattering times F_{τ}.

S3: Temperature coefficient of platinum conduction lines

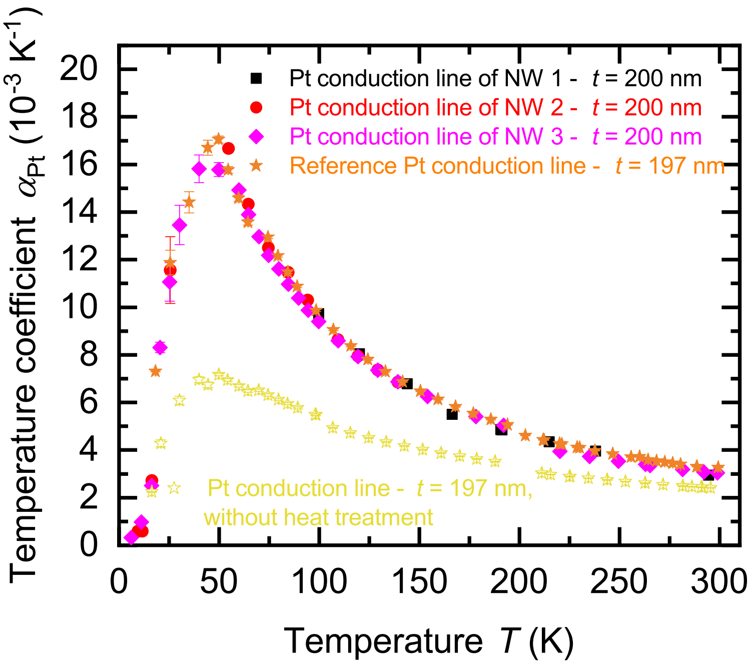

FIG. 2: **Temperature coefficient of platinum conduction lines.** Temperature coefficient of the resistance of platinum conduction lines α_{Pt} versus bath temperature T.

We ensured that the platinum conduction lines were equivalent by using the same purity of the sputtering material, by the measure of the thickness, by the same heat treatment, by the same residual resistance ratio and by the same temperature coefficient of the resistance. For these reasons, the conduction lines that were used in the present work as Seebeck reference material for the silver nanowires and the platinum conduction lines that were used in the separate experiment[2] can be seen as identical from a thermoelectric point of view.

Figure 2 shows the temperature coefficient of the resistance of the platinum conduction lines α_{Pt} that were used for the relative Seebeck measurements of the silver nanowires (samples NW 1 - NW 3) as a function of the bath temperature T. Furthermore, the temperature coefficient of the reference platinum conduction line, whose absolute Seebeck coefficient was used to determine the absolute Seebeck coefficient of the silver nanowires, is given. The absolute Seebeck coefficient of this platinum conduction line was determined in a separate experiment[2]. These temperature coefficients are all in agreement with each other. In addition, the temperature coefficient of a platinum thin film with the same thickness but without additional heat treatment is added. The lack of the heat treatment leads to a temperature coefficient that is clearly reduced compared to the reference platinum conduction line. This in turn leads to a reduced absolute Seebeck coefficient compared to the thin film with heat treatment. A detailed discussion of the absolute Seebeck coefficient of thin platinum films and the effects of heat treatment on the transport properties is given in reference[2].

[1] Smith, D. R., Fickett, F. R. Low-temperature properties of silver. *Journal of Research of the National Institute of Standards and Technology* **100**, 119 (1995).

[2] Kockert, M., Mitdank, R., Zykov, A., Kowarik, S., Fischer, S. F. Absolute Seebeck coefficient of thin platinum films. *Journal of Applied Physics* **126**, 105106 (2019).
